# A Novel Anthropomorphic Phantom Composed of Tissue-Equivalent Materials for Use in Experimental Radiotherapy: Design, Dosimetry and Biological Pilot Study

**DOI:** 10.3390/biomimetics8020230

**Published:** 2023-05-31

**Authors:** Thomas Breslin, Jason Paino, Marie Wegner, Elette Engels, Stefan Fiedler, Helen Forrester, Hannes Rennau, John Bustillo, Matthew Cameron, Daniel Häusermann, Christopher Hall, Dieter Krause, Guido Hildebrandt, Michael Lerch, Elisabeth Schültke

**Affiliations:** 1 Department of Oncology, Clinical Sciences, Lund University, 22185 Lund, Sweden; 2 Centre of Medical Radiation Physics, University of Wollongong, Wollongong 2522, Australia; 3 Institute of Product Development and Mechanical Engineering Design, Hamburg University of Technology, 21073 Hamburg, Germany; 4 Australian Synchrotron/ANSTO, Clayton 3168, Australia; 5 European Molecular Biology Laboratory (EMBL), Hamburg Outstation, 22607 Hamburg, Germany; 6 School of Science, Royal Melbourne Institute of Technology (RMIT) University, Melbourne 3001, Australia; 7 Department of Radiooncology, Rostock University Medical Center, 18059 Rostock, Germany

**Keywords:** anthropomorphic phantom, CT scan, dosimetry, experimental radiotherapy, microbeam radiotherapy (MRT), tissue-equivalent materials, therapy planning

## Abstract

The production of anthropomorphic phantoms generated from tissue-equivalent materials is challenging but offers an excellent copy of the typical environment encountered in typical patients. High-quality dosimetry measurements and the correlation of the measured dose with the biological effects elicited by it are a prerequisite in preparation of clinical trials with novel radiotherapy approaches. We designed and produced a partial upper arm phantom from tissue-equivalent materials for use in experimental high-dose-rate radiotherapy. The phantom was compared to original patient data using density values and Hounsfield units obtained from CT scans. Dose simulations were conducted for broad-beam irradiation and microbeam radiotherapy (MRT) and compared to values measured in a synchrotron radiation experiment. Finally, we validated the phantom in a pilot experiment with human primary melanoma cells.

## 1. Introduction

Tumor cell destruction, and normal-tissue preservation and structural and functional regeneration determine the quality of life in cancer patients undergoing radiotherapy. To predict the treatment outcome in radiation oncology, it is important to correlate the measurements of technical dosimetry to the biological response in both tumor and healthy tissue. While the response of the tumor tissue will inform about the dose required to reduce the tumor size or, if possible, to completely destroy the primary tumor, the response of the healthy tissue in the path of the treatment beam or in the immediate tumor environment indicates the normal-tissue tolerance. Normal-tissue tolerance is closely associated with the risk of unwanted adverse effects of radiotherapy and thus determines the upper dose limit for the target dose. In clinical radiooncology, X-ray dose measurement is conducted using ionization chambers or microDiamond detectors for absolute dosimetry and self-developing radiochromic film for relative dosimetry. The advantage of film dosimetry over absolute dosimetry is that, in addition to the dose deposited, it also records the geometry of the dose distribution. In the case of multiple irradiation fractions separated by constant time intervals, the doses registered on radiochromic film would be added in a mathematically linear fashion. In other words, the total dose to be read out after a series of irradiation fractions would depend very little on the dose deposited per fraction, on the duration of the interval between fractions or on the dose rate. Variation of those parameters will, however, significantly modify the biological response. The Timmerman tables illustrate how, in a clinically established technique called stereotactic radiotherapy, the variation of dose per fraction modifies normal-tissue response and how this is taken into account in the treatment planning [1]. In 2014, the tissue-preserving effect of broad-beam X-ray irradiation at high dose rates (>40 Gy/s, compared to typical clinical dose rates of 6–20 Gy/min) was brought to the attention of the radiooncology community [2]. The good preservation of normal-tissue function in microbeam radiotherapy (MRT), an experimental high-dose-rate radiotherapy approach using spatial dose fractionation at the micrometer range, has been well-known to the research community already for more than two decades [3]. 

Although the technically measured X-ray dose values might be equal after the administration of nominally equal doses of high-dose-rate and low-dose-rate broad-beam radiotherapy, the biological outcome would be significantly different [4]. Even more difficult is the comparison between broad-beam irradiation and spatially fractionated beam. The spatial dose fractionation causes a serial pattern of high-dose zones and low-dose zones in the irradiation target. Technically, the dose administered across the entire irradiation target can be calculated and measured, representing the integrated dose. However, the biological effects will differ from those seen after irradiating an equally sized target in the broad-beam technique with a dose nominally equal to this integrated dose. Therefore, it is important to develop tools which allow a high throughput of samples for the assessment of biological effects produced in novel radiotherapy techniques, also in comparison to already clinically established radiotherapy. Phantoms simulating both the shape and the material composition of human tissue provide a good environment for this type of biological study.

Additive manufacturing processes (AM) of phantoms using tissue-equivalent materials are at the core of this development and have been discussed before [5,6,7,8]. They allow the reliable reproduction of a standardized biological environment suitable to obtain replicates of biological data to obtain statistical power, aiming to answer a specific set of questions. AM can be used as a direct or indirect manufacturing process, mimicking biological properties on the one hand and creating anatomically geometric shapes on the other [9]. In modern clinical radiotherapy, the treatment plan is most frequently generated based on both the gross morphology and on attenuation coefficients represented in Hounsfield units (CT numbers). Thus, to design a phantom useful for experimental radiotherapy, knowledge of the CT numbers is important to make a choice of the materials best suited to represent the biological tissues [5]. 

In preparation of future clinical trials, we designed and produced a partial upper arm phantom using an additive manufacturing process as well as tissue-equivalent materials. This phantom is suitable for both technical dosimetry and the insertion of biological samples, allowing the correlation of nominal X-ray doses and biological effects. We have validated this phantom in a pilot study conducted with high-dose-rate irradiation techniques at the Imaging and Medical Beamline (IMBL) of the Australian Synchrotron.

## 2. Materials and Methods

The phantom produced is designed to simulate the anatomical and radiological characteristics of a human upper arm, making it an ideal model for testing and optimizing radiation therapy treatments. The sequence of processes includes data acquisition from an anonymized human patient CT scan, the segmentation of structures as basis for the design process, editing and slicing of the designed model for better visualization and the additive manufacturing from tissue-equivalent materials. In several postprocessing steps, the phantom was adapted to the needs of the current experiment. In a pilot experiment, radiobiological data were obtained at the Imaging and Medical Beamline (IMBL) of the Australian Synchrotron. 

### 2.1. CT Image Acquisition

**Figure 1:**
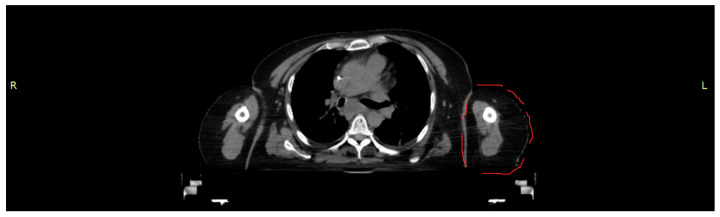
CT scan used as template for the partial upper arm phantom (section outlined in red).

A CT scan of a female torso, acquired on a Brilliance CT Big Bore Oncology scanner (Philips, Hamburg, Germany) operating at 290 mA and 120 kV, was used to obtain the data for both morphology and density values as the basis for an anthropomorphic phantom representing part of the left upper arm (Figure 1). Based on density (CT numbers), the images were segmented in 3D Slicer (Alliance for Medical Image Computing (NA-MIC), Boston, MA, USA) into tissue classes corresponding to skin, cortical bone, bone marrow and soft tissue. The segmented tissues were exported as Standard Transformation Language (STL) files.

### 2.2. Design and Production of a Partial Upper Arm Phantom

**Figure 2:**
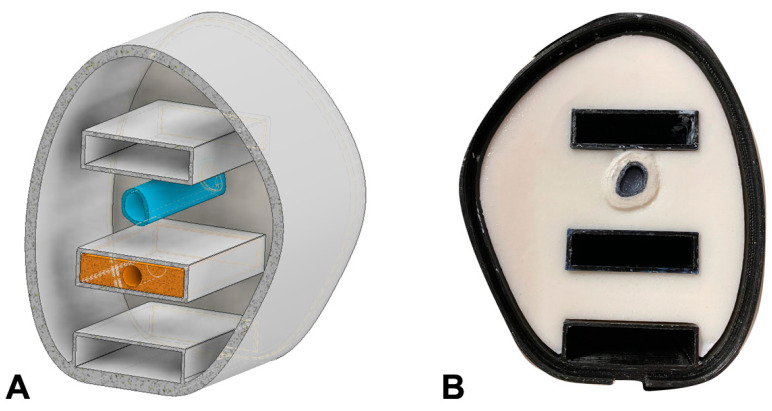
CAD model of the partial upper arm phantom (**A**) and axial view of the 3D printed phantom (**B**).

After data acquisition and segmentation, the STL files were imported into the CAD software Autodesk Inventor (Autodesk Inventor, Autodesk Inc., San Rafael, CA, USA). The arm phantom was designed as a hollow body where other surrogates can be filled in after 3D printing (see Figure 2A). Three compartments were designed to integrate dosimeters at different positions inside the arm phantom.

The outer shell of the phantom was additively manufactured using a fused deposition modeling (FDM) process (printer Anycubic i3 Mega S, Shenzhen Anycubic Technology Co., Ltd., Shenzhen, China), which allows for a high degree of accuracy and detail in the final product. Segmentation for printing and the generation of the g-code was prepared with Ultimaker Cura (Ultimaker Cura, Ultimaker, Utrecht, The Netherlands). For the printing material, a black polyethylene terephthalate (PETG) was chosen, due to its heat resistance and good printing quality. The shell is designed to mimic the shape and contours of a human upper arm segment, with precise anatomical measurements and proportions. The inner compartments of the shell are filled during post-processing with a soft tissue and bone equivalent.

The bone equivalent was designed to represent both the cortical long bone and the bone marrow of the arm, thus providing a complete simulation of the arm’s anatomy. The cortical bone is represented by plaster bandages consisting of gypsum (CaSO_4_) which are applied with water to the outside of the printed hollow bone cylinder. After drying, the gypsum was also coated with clear lacquer to prevent the penetration of moisture. The bone marrow inside the cylinder was simulated by a mixture of Vaseline (75 wt.%) (Carl Roth GmbH + Co. KG, Karlsruhe, Germany) and dipotassium hydrogen phosphate (K_2_HPO_4_) (25 wt.%). 

To create the soft tissue surrogate, materials with similar electron densities and CT numbers, compared to human tissue, were used. This ensures that the phantom behaves similarly to real tissue when exposed to ionizing radiation. A mixture composed of water (94.5 wt.%), agarose (2 wt.%), carrageenan (1.5 wt.%) and CaCO_3_ (2 wt.%) (Carl Roth GmbH + Co. KG, Karlsruhe, Germany) served as soft tissue surrogate. This soft tissue surrogate was mixed while heated and then carefully filled into the additive-manufactured shell to create a realistic representation of the arm tissue (see Figure 2B). 

Furthermore, the phantom is equipped with different inserts which allow for precise measurement of radiation dose. These inserts can accommodate radiochromic film and dosimeters used in clinical radiotherapy (ion chambers or microDiamond probes) at three different positions to monitor the radiation exposure of the phantom. They also allow insertion of tubes filled with biological samples in the same position as the microDiamond probes. The custom-made inserts are crafted from sheets of Polymethyl Methacrylate (PMMA), commonly known as acrylic (physical density $=1.19\;g/{cm}^{2}$) with a 7 mm hole to accommodate either a PTW microDiamond detector (TM 60019, PTW, Freiburg, Germany) or vials for cell samples (NMR glass tube (TeachSpin, Buffalo, NY, USA).

### 2.3. Imaging of the Upper Arm Phantom and Comparison with the Original CT

The phantom CT images were acquired at the same CT scanner as the original CT (Philips Brilliance Big-Bore, Hamburg, Germany), serving as the basis for the phantom design, collecting 1 mm thick slices with a lateral resolution of 0.61 mm in x- and y-directions. The original patient CT had a slice thickness of 3 mm and a lateral resolution of 1.37 mm. 

Using RayStation software (RaySearch, Stockholm, Sweden), the densities of the phantom generated from tissue-equivalent materials were compared to those obtained in the original CT scan. For plotting, the ‘CT to density definition’ was extracted from RayStation software to calculate horizontal density distributions of the patient and the phantom.

### 2.4. Dose Simulation and Measurement

Reference dosimetry was performed in a 100 mm × 100 mm × 100 mm Perspex^TM^ solid water phantom using a PTW PinPoint 3D Ionization Chamber (31022, Freiburg, Germany). With a sensitive volume of 0.016 $cm^{3}$, the PinPoint IC is certified for broad-beam field sizes as small as $20\times 20\;mm^{2}$, making it the ideal candidate for small field and small animal irradiation [10]. The entrance dosimetry protocol used for this work was developed at DESY (Hamburg, Germany), and is adapted from that used at the ESRF (Grenoble, France) [11]. The entrance dose was defined at an equivalent measurement depth of 5 mm. Here, the measurement was performed in the DESY entrance reference phantom, which is a 50 mm × 50 mm × 50 mm Perspex^TM^ phantom using the PTW PinPoint 3D IC, with the central axis of the IC at 5 mm depth.

For microbeam characterization, the PTW microDiamond (60019, Freiburg, Germany) detector was cross-calibrated to the PTW PinPoint chamber under the same entrance reference phantom measurement conditions. With a minimal cross-sectional area of $1.1\times 0.001\;mm^{2}$ the PTW microDiamond is ideal for microbeam characterization [12,13]. 

Geant4 dose simulations were performed using the previously validated DoseMRT software package (accepted for publication to MDPI Physics 2023). The software package was used to import a CT scan of the 3D printed arm phantom and convert it into a voxelized phantom with custom defined materials based on the known composition of the phantom. Experimental dose measurements in the surface reference phantom were used to calibrate simulation results. 

### 2.5. Melanoma Cell Culture

Human primary melanoma cell cultures, derived from a secondary melanoma without BRAF V600E mutation (D24, isolated by N. H. at the Queensland Institute of Medical Research), were used for this proof-of-principle experiment to simulate metastatic spread in different tissue depths. In a standard incubator (37 °C, 5% CO_2_), cells were raised in RPMI 1640 medium GlutaMAX supplement (61870036, Gibco, Life Technologies, USA) supplemented by 10% fetal bovine serum and 1% (*v*/*v*) Penicillin–streptomycin–glutamine (catalogue number 10378016, Gibco, Life Technologies, USA) to approx. 70% confluence, and harvested using 0.05% Trypsin 5 to 10 min at 37 °C (until the cells detached). 

Agar (catalogue number 4508.1, Carl Roth GmbH, Karlsruhe, Germany) was heated to below boiling point in sterile filtrated water, prepared at a concentration of 1%. The clear agar suspension was allowed to cool down and mixed with the melanoma cells to achieve a cell concentration of approx. 1 million cells per ml in a final agar concentration of 4%. They were apportioned for the experiment in 3D printed tubes from polylactic acid+ (PLA+) polymer filament (eSun, Shenzhen, China), where they solidified at room temperature for 1 h. Prior to the cell culture experiment, the material was tested in cell cultures and found not to impair cell proliferation. The suspension of the cells in agar allowed a three-dimensional distribution of the cells in a larger volume for the purpose of this irradiation experiment. For the experiment, in each of six NMR glass tubes (7 mm diameter, TeachSpin, Buffalo, NY, USA), 200 µL cell suspension in agar was suspended in 800 µL of clear growth medium (without pH indicator).

### 2.6. Synchrotron Irradiation of Melanoma Cell Samples

**Figure 3:**
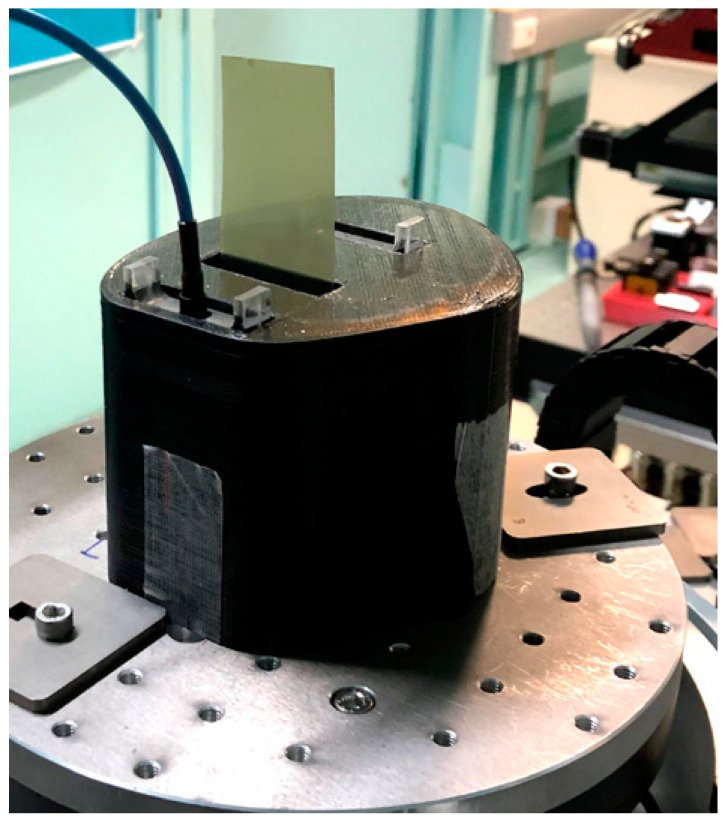
Experimental setup of the partial upper arm phantom with the microDiamond probe in the position close to beam entry and self-developing radiochromic film in the middle insert slot, compact dummy insert module in the third insert slot, at IMBL Hutch 2B.

Dose measurement and irradiation were conducted in Hutch 2B of the IMBL of the Australian Synchrotron at 124.09 keV and a ring current of 200 mA. The phantom was set up to simulate medial-to-lateral irradiation of the arm (Figure 3). The correlating positioning would be that of a patient lying on his back with his arm raised at an angle of 90°, with the medial aspect of the arm facing the beam. Gafchromic™ film at beam entrance and exit positions was used to verify the beam geometry and the fact that the entire sample had been irradiated. All three positions in the inserts were filled, in subsequent irradiation exposures, with samples to assess the biological effects of depth-dependent dose attenuation. During each of these measurements, the other two slots were filled with the plexiglass inserts.

The beam height at the treatment position was 2.01 mm. The phantom was moved vertically through the beam at a speed of 0.128 mm/s. Microbeam irradiation was conducted with MRT peak doses of 400 Gy and respective valley doses at skin entrance, delivered in an array of quasi-parallel microbeams where the individual microbeam width was 50 µm and the center-to-center distance was 400 µm. 

### 2.7. WST Test

After the irradiation, the samples were placed into a standard incubator for approx. two hours. Then, the agar-cell suspension was homogenized carefully in the growth medium using a 200 µL pipette tip. The number of cells per sample was counted and approx. 5000 melanoma cells per well were seeded in 96-well plates in triplicate for each sample. The volume in each well was topped up to 100 µL per well with growth medium, after which the well plates were placed into the standard incubator. 

The samples were analyzed using a commercially available WST test (catalogue number 05015944001, Roche Diagnostics GmbH, Mannheim, Germany) at 48 and 72 h after irradiation. The WST test provides a simple and accurate method to assess cell proliferation. The reagent detects the cleavage of the tetrazolium salt WST-1 (4-[3-(4-Iodophenyl)-2-(4-nitro-phenyl)-2H-5-tetrazolio]-1,3-benzene sulfonate) to formazan by mitochondrial dehydrogenase in a one-step procedure. An increase in viable cells is reflected in an increase in formazan dye production. The absorbance of the formazan dye can be detected at a wavelength range 420–480 nm. Due to the specifics of the available plate reader (Muse Biotek Synergy, Agilent, Santa Clara, CA, USA), we chose to read the absorbance at 450 nm, with a reference wavelength of 620 nm.

To each well with sample suspended in 100 µL growth medium, 10 µL of reagent were added. In addition, 10 µL of sample was added to 100 µL of growth medium without samples, serving as blank controls. The well plates were returned to the incubator for 2 h before readout, which was performed at 48 and 72 h after irradiation.

A non-parametric one-way ANOVA test (GraphPad Prism 6, GraphPad Software, Inc., La Jolla, CA, USA) was used to tabulate the blank-corrected absorption values and assess the statistical significance of the data.

## 3. Results

### 3.1. Comparison of Structures in Phantom and Original CT

The density values for the main tissue components (bone, bone marrow and soft tissue) were extracted from the CT scans obtained from both the phantom and the original patient using RayStation software (RaySearch, Stockholm, Sweden). The density variations were within 7% between the phantom and the original for all three components (Table 1). 

**Figure 4:**
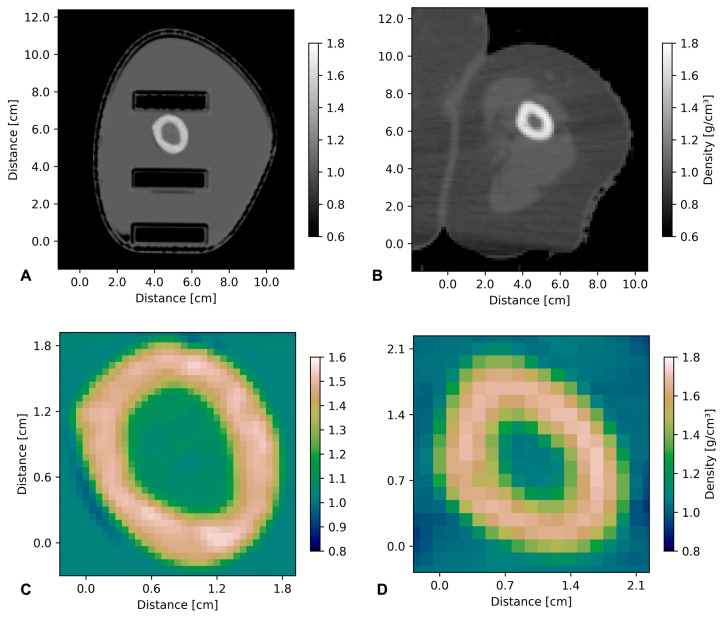
Comparison of density values between phantom CT (**A**,**C**) and original patient CT (**B**,**D**). Upper panel: CT images of phantom (**A**) and original CT (**B**). Lower panel: zoom on the area around the bone. In accordance with the numerical values, the density values are very similar in both CT scans. This is important because density values of different tissues/compartments are used as the basis for medical physics planning in modern radiotherapy. Inhomogeneities within the bone are seen in both the phantom and the human original CT.

Choosing a representative region containing all three tissue components, density maps were created of the area around the bone structures (Figure 4).

### 3.2. Dose Simulations and Measurements

**Figure 5:**
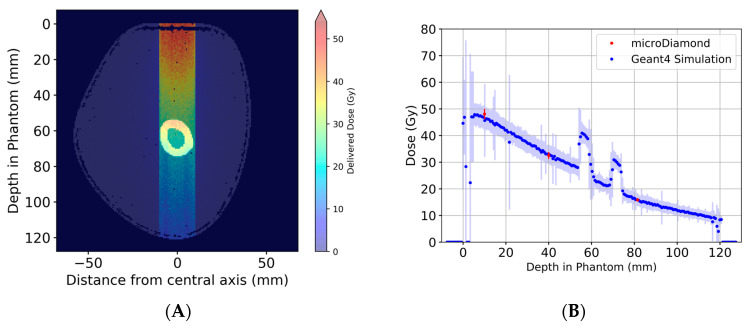
Geant4 simulations of broad-beam irradiation of arm phantom. The 2D dose distribution across the central axis of the beam is shown in (**A**). The dose at the center of the beam for each depth in the phantom, good agreement between Geant4 simulations and experimental dosimetry using the TPW microDiamond detector (**B**). The peaks at approx. 55 mm and 70 mm depth are due to the bone structures, while the material in between the bone structures, the bone marrow, has a density very similar to that of soft tissue. This is true for both the phantom and the patient data sets (see also Figure 4C,D).

In the following example, a dose was prescribed as entry dose, here defined as 5 mm depth in an acrylic reference phantom with dimensions 40 × 440 × 23 mm (W × H × D). Dosage was also recorded at the depth of the three inserts for a surface prescription dose of 50 Gy. The doses recorded at the three insert positions were 48.10 Gy at 10 mm, 32.51 Gy at 40 mm and 15.85 Gy at 81.5 mm from the beam entry surface (Figure 5A).

Dosimetry was performed for the reference dosimetry phantom only and again used to calibrate Geant4 simulations for all depths. The variation in the reproducibility of the experimentally measured entrance dose is 1% the uncertainty in the measured absolute dose is greater of course, being 7%. Table 2 below summarizes the broad-beam and microbeam peak doses for each irradiation modality. The uncertainty quoted in Table 2 represents the average over all depths simulated. 

### 3.3. Pilot Experiment Tumor Cell Destruction

**Figure 6:**
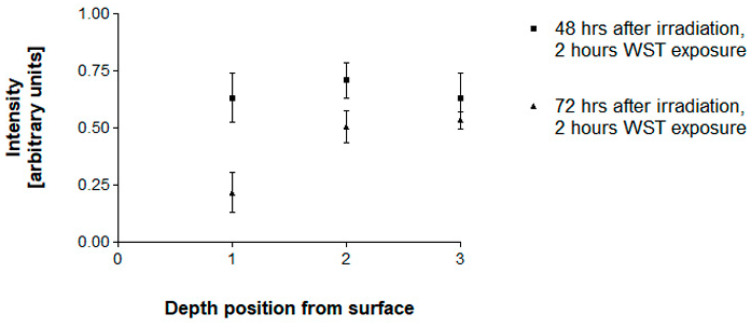
Dose-dependence of tumor cell destruction. At position 1 (10 mm from the surface, exposed to the highest irradiation dose), cell death is increased between days 2 and 3 after MRT with peak doses of 400 Gy by another 46%. Deeper in the tissue, at position 3 (81.5 mm from the surface, exposed to the lowest irradiation dose), the additional increase in cell destruction is only 16%. The error bars are SEM.

Due to the dose attenuation increasing with depth from surface, the extent of tumor cell destruction determined at 72 h after irradiation decreases with depth from surface (Figure 6). Interestingly, the measured values at 48 h after irradiation are almost equal at all three depth points. Only the values obtained at 72 h after irradiation are distinctly depth-dependent. A possible explanation would be that, at 48 h after irradiation, cell death is still mainly the result of necrotic cell death due to the high microbeam peak doses to which the cells were exposed, even at a depth of approx. 8 cm below the surface (Table 2). At 72 h after irradiation, however, we are looking at an effect already strongly modulated by the lower valley dose, bystander effects in the non-directly irradiated region between the microbeams. The valley dose of approx. 8 Gy at the sample position closest to the surface elicits a strong tumor-destructive response. With increasing depth from the surface, this dose decreases rapidly. At 8 cm depth, the valley dose contributes significantly less to additional tumor cell destruction.

## 4. Discussion

The need for anatomically correct phantoms (anthropomorphic phantoms) has been previously emphasized for the optimization of imaging processes [14]. For simulation purposes in experimental radiotherapy, this need has also been recognized. Anthropomorphic phantoms are commercially available, with a varying emphasis on tissue equivalence [15,16]. However, these phantoms are expensive, and, in our experience, it is difficult-to-impossible to obtain specific parts of them in duplicate or triplicate to facilitate high-throughput experiments. 

We have, therefore, custom-designed and produced an anthropomorphic phantom from materials determined to have comparable qualities for the purposes of imaging and radiotherapy. We found that this tissue-equivalent phantom of a human upper arm provides an anatomically highly accurate, realistic model for testing and optimization in experimental radiotherapy. It is suitable for clinically relevant dose simulation as well as for in vitro radiobiology studies. In dosimetry studies for high-dose-rate broad-beam and spatially fractionated radiotherapy, it allows for precise dose measurements and thus provides a valuable tool for medical research and treatment planning. As an advantage over commercially available phantoms, custom-printed insert modules allow the insertion of dosimeters and biological samples generated from human tumor cell lines in the same position, thus permitting a direct correlation between absorbed X-ray doses and the associated biological effects.

A further advantage is the relatively low material cost (less than EUR 50) associated with the additive manufacturing process and the pouring of the surrogates used. Additionally, the efficient printing and post-processing times ensure that the entire manufacturing process of the phantom takes only two days. Using AM allows for the creation of highly customized and precise models which can accurately replicate the complex geometries and structures of a human arm. Finally, 3D printing also allows for easy modifications to the design of the phantom model, enabling quick adjustment and optimization of the model for specific research or clinical applications.

Thus, besides its usefulness in the preparation of future clinical studies, we have demonstrated several important aspects of this phantom: it is inexpensive to produce and can be redesigned on short notice to accommodate different variants of the radiobiology experiment, for instance side-to-side sample duplicates or triplicates. Instead of tumor cell samples, normal cell samples could be inserted or, even more closely mimicking the normal microenvironment of the tumor, 3D co-cultures of tumor and normal cells.

The irradiation parameters are sufficiently stable to assume that differences in the cell statistics are not caused by variation of the irradiation parameters within the short time required to exchange the samples inside the phantom. However, to achieve a sufficiently high sample throughput within a restricted time window, the side-by-side positioning of several biological samples would significantly shorten the experimental time required to conduct this type of experiment. 

## 5. Conclusions

We have demonstrated the feasibility of constructing life-size anthropomorphic phantoms using tissue-equivalent materials, reproducing the three main tissue components of the human upper arm. In our study, the phantom was customized to conform to the demands of experimental radiotherapy, where it represents a vital tool in the evaluation of biological effects in conjunction with technical dosimetry, especially in the validation of novel radiotherapy techniques that cannot be extrapolated from conventional irradiation schedules, such as broad-beam FLASH and microbeam radiotherapy. The utilization of anthropomorphic phantoms to assess the concurrence between the planned and actual dose delivery of brings us one step closer to the clinical translation. 

The phantom can be easily customized to study normal cell behavior as well as tissue regeneration and repair processes following radiotherapy.

## Figures and Tables

**Table 1:** Overview densities units, measured in phantom and original CT.

	Phantom	Original CT
Bone	1.44–1.52 g/cm^3^	1.44–1.72 g/cm^3^
Bone marrow	1.04–1.1 g/cm^3^	1.06–1.13 g/cm^3^
Soft tissue	1.03–1.04 g/cm^3^	0.94–1.08 g/cm^3^

**Table 2:** Surface dose based on experimental measurements in reference phantom. Dose at depth reported from Geant4 simulation results.

Modality	Entrance Dose (±7%)	Dose at Depth 1 (10 mm) (±8%)	Dose at Depth 2 (40 mm) (±8%)	Dose at Depth 3 (81.5 mm) (±8%)
Broad beam	50 Gy	48.10 Gy	32.51 Gy	15.85 Gy
Broad beam	400 Gy	384.8 Gy	260.08 Gy	126.8 Gy
Microbeam Peak	50 Gy	47.25 Gy	34.02 Gy	17.78 Gy
Microbeam Peak	400 Gy	378.02 Gy	272.16 Gy	142.24 Gy

## Data Availability

Data are available upon request from the corresponding author (E.S.).
